# Forced vaginal sex and genital immune correlates of HIV risk: a prospective study of female sex workers in Kenya

**DOI:** 10.1136/bmjgh-2024-018219

**Published:** 2025-12-19

**Authors:** James Pollock, Mary Kung’u, Suji Udayakumar, Sanja Huibner, Rhoda Kabuti, Hellen Babu, Erastus Irungu, Polly Ngurukiri, Peter Muthoga, Wendy Adhiambo, Helen A Weiss, Janet Seeley, Tanya Abramsky, Joshua Kimani, Tara S Beattie, Rupert Kaul, Maisha Fiti Study Champions

**Affiliations:** 1 Immunology, University of Toronto, Toronto, Ontario, Canada; 2 Partners for Health and Development in Africa, Nairobi, Kenya; 3 Medicine, University of Toronto, Toronto, Ontario, Canada; 4 Infectious Disease Epidemiology and International Health, London School of Hygiene & Tropical Medicine, London, UK; 5 Global Health & Development, London School of Hygiene & Tropical Medicine, London, UK

**Keywords:** Kenya, Interdisciplinary Research, HIV, Global Health, Gender-Based Violence

## Abstract

**Background:**

The likelihood of HIV acquisition is increased following forced vaginal sex. This relates in part to epidemiological and behavioural factors; however, the biological effects of forced vaginal sex, including impacts on immune parameters linked to HIV susceptibility, are poorly understood. Here, we examine biological mediators of HIV susceptibility among female sex workers (FSWs) in Nairobi, Kenya, who recently experienced forced vaginal sex.

**Methods:**

The Maisha Fiti study was a longitudinal cohort study of FSWs from Nairobi, Kenya. At up to three visits, HIV-uninfected participants completed a detailed sociodemographic survey in which they were asked if they had experienced forced vaginal sex in the past 7 days. Proinflammatory cytokines and soluble E-cadherin (sE-cad), a biomarker of epithelial barrier disruption, were quantified in cervico-vaginal secretions by multiplex immunoassay. Associations between recent forced sex and genital inflammation were assessed longitudinally in a mixed-effects regression model adjusted for potential confounders and within-participant correlation.

**Results:**

Of the 746 participants, 44 (6%) reported forced vaginal sex in the past 7 days at baseline, with strong evidence of associations with adverse childhood experiences (p<0.001), mental health issues (p<0.001) and poverty (p=0.02). Recent forced sex was associated with increased genital inflammation (adjusted OR (aOR)=2.74; 95% CI 1.33 to 5.68; p<0.01) independent of previously defined confounders but was not associated with altered levels of sE-cad (p=0.56). Neither recent consensual sex (aOR=0.94, 95% CI 0.63 to 1.40, p=0.76) nor forced sex within the past 6 months, excluding the past 7 days (aOR=0.93, 95% CI 1.21 to 5.42, p=0.70), was associated with genital inflammation.

**Conclusions:**

Cervicovaginal inflammation is increased in FSWs for at least a week after forced vaginal sex. This has important implications for HIV prevention programmes that provide care to women experiencing gender-based violence. Further studies are needed to understand the specific timing of proinflammatory cytokine release following forced vaginal sex.

**WHAT IS ALREADY KNOWN ON THIS TOPIC**

**WHAT THIS STUDY ADDS**

- When we measure soluble immune factors in female sex workers in Nairobi, Kenya, we find that those who self-report forced vaginal sex in the past 7 days are more likely to have genital inflammation, which increases the likelihood of acquiring HIV the next time they are exposed to the virus. These women were also more likely to be experiencing poverty and mental health issues and, on average, met with multiple clients in the same week they were sexually assaulted.

**HOW THIS STUDY MIGHT AFFECT RESEARCH, PRACTICE OR POLICY**

- Further integration of HIV and gender-based violence services and enhanced pre-exposure prophylaxis and postexposure prophylaxis access for women who experience sexual violence are urgently needed to address the intersectional challenges that women exposed to HIV and violence face.

## Introduction

 Sexual assault is a form of gender-based violence (GBV) experienced by 6% of women globally at least once in their lifetime^1^ and has both significant short-term impacts such as injury, undesired pregnancy and death, as well as longer-term consequences for physical and mental health.^2^ Estimates of global past-year incidences of sexual assault differ depending on the definition of sexual assault used, but regional incidences are high even when restrictive definitions of sexual assault (forced penile/vaginal penetration)^3^ are used. In regions with high HIV prevalence among the general population, women exposed to forced vaginal sex also face an increased risk of acquiring HIV.^4^

Men who perpetrate GBV are more likely to be living with HIV,^5^ and more likely to report sexually transmitted infection (STI) symptoms and engage in multiple and concurrent sexual partnerships, transactional sex and substance use.^6^ Additionally, women exposed to violence may exhibit trauma-induced behavioural changes that increase HIV risk.^6^ These include an increased likelihood of heavy substance/alcohol use, engagement in commercial sex and engagement in multiple and concurrent sexual partnerships, and a reduced likelihood of condom use.^6 7^ Therefore, there are numerous epidemiological and behavioural mechanisms through which forced vaginal sex may enhance HIV acquisition risk.

It is less clear if forced vaginal sex is a biological risk factor that directly enhances mucosal HIV susceptibility, that is, the ‘per contact’ risk of HIV acquisition after exposure. Overall HIV acquisition risk after a single episode of condomless receptive penile-vaginal sex with a treatment-naïve male partner is approximately 1/500, but this risk may increase in certain contexts.^8^ An important driver of vaginal HIV susceptibility is genital inflammation, which acts through two pathways. First, the inflammatory response initiates the recruitment of immune cells, including activated CD4+T cells that constitute a primary HIV target cell.^9^ While neutrophils produce soluble immune factors that have in vitro anti-HIV activity, their production of chemoattractant chemokines such as IL-8 enhances the recruitment of highly HIV-susceptible Th17 cells.^10^ Second, genital inflammation leads to disruption of the genital epithelium^11^ via several mechanisms: interactions with genital bacteria,^12^ direct effects of inflammatory cytokines on the genital epithelium,^13^ and through direct physical disruption.^14^ In keeping with this, genital inflammation in South African women, defined based on vaginal proinflammatory cytokine elevation, has been strongly associated with HIV acquisition.^15^

Forced vaginal sex exposure might cause genital inflammation and/or epithelial barrier disruption, but few studies have been able to directly address this hypothesis, partially due to small sample sizes and the challenges involved in sexual violence research.^16^ While the risk of genital injury is higher during non-consensual sex as compared with consensual sex,^17 18^ even consensual sex has important short-term immune effects, increasing vaginal levels of soluble E-cadherin (sE-cad), MIP-1β, MIG and IP-10, as well as the number of Th17 cells, activated CD4+T cells and activated DCs in the cervical epithelium for up to 48 hours.^19^ Forced vaginal sex may cause epithelial barrier disruption directly, due to tears in the epithelium,^18 19^ although evidence supporting this hypothesis is lacking. In addition, past violence exposure has been associated with decreased expression of genes involved in epithelial barrier homeostasis.^20^

In a large prospective study among female sex workers (FSWs) from Nairobi, Kenya, we now examine the associations between forced vaginal sex in the past 7 days and vaginal biomarkers of HIV susceptibility. FSWs are disproportionately affected by both HIV and GBV,^21^ and in a recent survey of FSWs in Nairobi, over half of the respondents reported sexual violence exposure in the past 12 months.^22^ We hypothesise that forced vaginal sex exposure in the past 7 days is independently associated with both: (1) genital inflammation, as defined by a composite score of cervicovaginal proinflammatory cytokines and (2) epithelial barrier disruption, as defined by elevated cervicovaginal levels of sE-cad.^14^

## Methods

### Study participants

The Maisha Fiti study was a longitudinal mixed-method study conducted from June 2019 to March 2021 that investigated biological, social and structural HIV risk factors and their biological impacts on genital inflammation. Participants constituted a random sampling of clients from seven sex worker outreach programme (SWOP) clinics across Nairobi. This paper uses data from three study visits: baseline (June–December 2019), midline (January–March 2020) and endline (June 2020–March 2021), with the midline visits interrupted by the onset of the COVID-19 pandemic. The characteristics of the cohort and study size determinations have been described previously.^23^ For the purposes of this analysis, only data from HIV-uninfected participants are used.

At up to three study visits, participants were asked to complete a behavioural questionnaire to collect sociodemographic data. Participants indicated recent forced vaginal sex exposure in the behavioural questionnaire by answering ‘Yes’ to the question: ‘Have you been physically forced to have sex in the past 7 days?’ (online supplemental table 1). The specific term ‘forced vaginal sex’ was used, rather than the umbrella term ‘sexual assault’, which can also refer to other forms of non-consensual sexual violence that are outside the scope of this study on vaginal immune impacts. A 7-day time frame was chosen in order to examine the impact of violence in the short term while having a large enough sample size to conduct our analysis.

Blood was collected for HIV diagnostics, and blood from HIV-uninfected participants was further tested for HSV-2 infection, syphilis infection and C reactive protein levels. Urine was collected for pregnancy testing, schistosomiasis diagnostics and *Chlamydia trachomatis* and *Neisseria gonorrhoeae* infection. Genital swabs were collected to test for bacterial vaginosis (BV) via Nugent scoring and *Trichomonas vaginalis*. Genital secretions were self-collected using a menstrual cup, transported on ice and cryopreserved at −80°C within 2 hours for subsequent soluble immune factor quantification (online supplemental materials 2).

### Quantification of soluble immune factors

Within 2 hours of each study visit, menstrual cups were transported to the Nairobi lab where they were placed in conical tubes and centrifuged at 555 g for 5 min. Samples were weighed and resuspended at a 10-fold dilution in sterile PBS before being centrifuged again at 1730 g for 10 min. Supernatant was frozen at −80°C and transported to Toronto for soluble immune factor measurement. In Toronto, samples were thawed at room temperature and centrifuged at 500 g for 5 min for immune factor analysis. Interleukin (IL)-1α, IL-1β, IL-6, IL-8, interferon gamma-induced protein (IP)-10, monocyte chemoattractant protein (MCP)-1, monokine induced by gamma interferon (MIG), macrophage inflammatory protein (MIP)-1α, MIP-1β, MIP-3α, tumour necrosis factor (TNF), sE-cad, and matrix metallopeptidase (MMP)-9 were measured in duplicate using the MSD platform, as previously described.^24^ Genital inflammation was defined by a binary composite score of inflammatory cytokines (IL-1α, IL-1β, IL-6, IL-8, IP-10, MCP-1, MIP-1α and MIP-1β) that has been associated with HIV seroconversion^15^ and subsequently employed in a previous study of the effect of past 12-month violence on genital inflammation in this cohort.^25^ Cytokines above the cohort median concentration threshold (based on the baseline concentration values) were considered ‘elevated’, and participants with at least 5 out of 9 elevated cytokines were defined as having genital inflammation. In cases of incomplete cytokine data, the genital inflammation status was assigned if the available data provided a robust classification, meaning that no plausible values for the missing cytokines could alter the status determination.

### Statistical analysis

Sociodemographic factors measured at the baseline study visit were explored among all HIV-uninfected Maisha Fiti participants in a descriptive analysis. We used chi-square tests and Welch’s t-tests to compare sociodemographic factors between participants exposed to forced vaginal sex in the 7 days preceding the baseline study visit and those not recently exposed. Sociodemographic analyses were weighted by age to account for oversampling of younger women.

Logistic mixed-effects regression models were constructed to explore the direct association between presence/absence of genital inflammation (a predefined coprimary endpoint) and self-reported forced vaginal sex exposure in the past 7 days, adjusting for potential confounders based on their known (or plausible) association with genital inflammation. All regression models were adjusted for age, socioeconomic status (SES), BV status by Nugent score, vaginal cleaning practices using soap, bacterial STI prevalence and high-risk substance use as defined by the WHO ASSIST tool. Data from all three visits was included in these models, and participant ID was included as a random effect to allow for the inclusion of correlated observations at all three time points without violating the assumption of independence.^26^ The effect of recent forced sex on log-transformed continuous sE-cad levels, the other coprimary study endpoint, was estimated using mixed effects linear regression and Kenward-Roger approximation to assess the significance of recent forced vaginal sex as a fixed effect. Additionally, we performed Welch’s t-tests to understand the individual cross-sectional associations between forced sex exposure and each log-transformed immune biomarker outcome at the baseline visit as exploratory secondary endpoints.

To compare genital inflammation with participants who had had consensual sex in the past 7 days at baseline, we defined three new participant groups: those who had not had any sex in the 7 days prior to the baseline study visit, those who had had only consensual sex in the 7 days prior to the baseline study visit, and those who had experienced any forced sex in the 7 days prior to the baseline study visit. Adjusted odds of genital inflammation were estimated in a cross-sectional logistic regression model.

To compare genital inflammation among participants with more remote (over a week ago) forced vaginal sex exposure at baseline, four new mutually exclusive groups of participants were defined: those who had never experienced forced sex, those who had only experienced forced sex more than 6 months before the baseline study visit, those who had experienced forced sex in the past 6 months but not the past 7 days, and those who had experienced forced sex in the 7 days prior to their baseline study visit. Adjusted odds of genital inflammation were estimated in a cross-sectional logistic regression model. All analysis was performed in R (V.4.3.1).

## Results

Overall, 746 HIV-uninfected Maisha Fiti participants completed the baseline questionnaire (June–December 2019). Of these, 265 (36%) participants attended the midline visit, and 637 (85%) attended the endline visit. At the baseline visit, the median age was 32 years (range 18–45 years). At baseline, participants met with a median of 4 clients in the past 7 days and had been engaged in sex work for a median of 8 years. 180 (32%) participants were taking postexposure prophylaxis (PrEP) at the time of the baseline study visit, and there were no HIV seroconversions during the course of the study.

In total, 44 (6%) HIV-uninfected Maisha Fiti participants reported forced sex in the past 7 days at baseline (table 1). These participants were more likely to be of lower SES (57% vs 31%; p<0.01) and to report 9 or more adverse childhood experiences (39% vs 15%; p<0.01), to be experiencing depression (55% vs 22%; p<0.001) and post-traumatic stress disorder (PTSD) (34% vs 13%; p=0.01) and to be a high risk substance user (48% vs 11%; p<0.001) than participants not reporting recent (past 7 days) forced sex. Age (median 32 vs 32; p=0.89), number of clients in the past 7 days (median 4 vs 4; p=0.82) and bacterial STI prevalence (11% vs 11%; p=0.69) did not differ between exposed and non-exposed groups, and there was evidence of a negative association between recent forced vaginal sex and BV prevalence (4% vs 18%; p<0.01). Only 3 (7%) of the participants who reported forced sex in the past 7 days had accessed HIV postexposure prophylaxis (PEP).

**Table 1:** Baseline sociodemographics of HIV-uninfected Maisha Fiti participants

		No forced vaginal sex, past 7 days (n=702)	Forced vaginal sex, past 7 days (n=44)	P value*
Age (median, range)		32 (18–45)	32 (20–45)	0.89
Low SES		216 (31%)	25 (57%)	**<0**.**01**
nine or more ACEs		103 (15%)	17 (39%)	**<0**.**01**
Number of clients, past 7 days (median, range)		4 (0–70)	4 (0–30)	0.82
Street-based client solicitation		198 (28%)	20 (45%)	0.08
Hormonal contraceptive use		138 (20%)	11 (25%)	0.57
Vaginal cleaning (soap)		415 (59%)	29 (66%)	0.76
Current mental health problem	Depression	151 (22%)	24 (55%)	**<0**.**001**
	Anxiety	68 (10%)	9 (20%)	0.11
	PTSD	90 (13%)	15 (34%)	**0**.**01**
Harmful substance use		76 (11%)	21 (48%)	**<0**.**001**
Bacterial STI††		81 (11%)	5 (11%)	0.69
Bacterial vaginosis		125 (18%)	2 (4%)	**<0**.**01**

* Welch’s t-tests were used to assess differences between groups for continuous variables, while χ2 tests were used to assess differences between groups for categorical variables. Bold values denote statistical significance at the 5% level.

† Chlamydia, gonorrhoea or syphilis infection.

ACEs, adverse childhood experiences; PTSD, post-traumatic stress disorder; SES, socioeconomic status; STI, sexually transmitted infection.

Genital inflammation data were available for 711 (95%) participants at baseline, including 39 of 44 participants who experienced forced sex in the past 7 days, overall, 351 (49%) participants demonstrated inflammation. Genital inflammation was present among 27 (69%) participants who experienced forced sex in the past 7 days, and 324 (48%) participants who did not recently experience forced sex.

In the longitudinal analysis, genital inflammation was strongly associated with recent forced sex (adjusted OR (aOR)=2.74, 95% CI 1.33 to 5.68; p<0.01), adjusted for age, SES, BV status, vaginal cleaning practices using soap, bacterial STI prevalence and high-risk substance use (table 2). In this model, intermediate Nugent score (aOR=1.89, 95% CI 1.33 to 2.67; p<0.001) and the detection of a prevalent bacterial STI (aOR=1.85, 95% CI 1.14 to 3.00; p=0.01) were also independently associated with genital inflammation, while high risk substance or alcohol use was associated with a reduced likelihood of genital inflammation (aOR=0.49, 95% CI 0.29 to 0.82; p<0.01).

**Table 2:** Associations between genital inflammation and forced vaginal sex exposure in the past 7 days (mixed-effects logistic regression model)

	Fixed effect estimate	aOR	95% CI	P value
Forced sex exposure, past 7 days	1.01	2.74	1.33 to 5.68	**<0.01**
Age	0.002	1.00	0.98 to 1.02	0.82
Medium SES	0 (reference)			
Low SES	−0.09	0.91	0.62 to 1.33	0.63
High SES	−0.09	0.92	0.63 to 1.33	0.65
Low Nugent score	0 (reference)			
Intermediate Nugent score	0.63	1.89	1.33 to 2.67	**<0.001**
High Nugent score	−0.30	0.74	0.48 to 1.14	0.17
Vaginal cleaning (soap)	−0.29	0.75	0.42 to 1.35	0.34
Bacterial STI*	0.61	1.85	1.14 to 3.00	**0.01**
Low substance use	0 (reference)			
Medium substance use	−0.19	0.83	0.59 to 1.15	0.26
High substance use	−0.71	0.49	0.29 to 0.82	**<0.01**

Bold values denote statistical significance at the 5% level.

* Chlamydia, gonorrhoea or syphilis infection.

aOR, adjusted OR; SES, socioeconomic status; STI, sexually transmitted infection.

Individual cytokines were then analysed as exploratory endpoints in an unadjusted cross-sectional analysis. Participants recently exposed to forced sex had higher vaginal levels of IL-6 (mean 1.46 vs 1.22 log_10_ pg/mL; p=0.04), MCP-1 (mean 2.04 vs 1.78 log_10_ pg/mL; p=0.02), MIP-1α (mean 1.60 vs 1.39 log_10_ pg/mL; p=0.04) and MIP-3α (mean 2.55 vs 2.30 log_10_ pg/mL, p<0.01) at baseline, compared with women not recently forced to have sex (online supplemental figure 1).

In adjusted analyses, there was no evidence that sE-cad was associated with reported recent forced sex (adjusted mean difference=0.03, p=0.54) (table 3). sE-cad levels were higher among those with an intermediate (p<0.001) or high Nugent score (p<0.001), but lower among those with lower SES score (p=0.048).

**Table 3:** Associations between soluble E-cadherin levels and forced vaginal sex exposure in the past 7 days (mixed effects linear regression model)

	Fixed effect estimate	SE	T test statistic	P value
Forced sex exposure, past 7 days	0.03	0.07	0.62	0.54
Age	−0.000003	0.002	−0.002	0.99
Medium SES	0 (reference)			
Low SES	−0.07	0.03	−1.98	**0.048**
High SES	0.01	0.03	0.33	0.737
Low Nugent score	0 (reference)			
Intermediate Nugent score	0.51	0.03	19.23	**<0.001**
High Nugent Score	0.63	0.03	21.12	**<0.001**
Vaginal cleaning (soap)	0.001	0.04	0.03	0.98
Bacterial STI*	0.007	0.04	0.19	0.85
Low substance use	0 (reference)			
Medium substance use	−0.02	0.03	−0.81	0.42
High substance use	−0.01	0.04	−0.30	0.79

Bold values denote statistical significance at the 5% level.

* Chlamydia, gonorrhoea or syphilis infection.

aOR, adjusted OR; SES, socioeconomic status; STI, sexually transmitted infection.

Having found strong associations between forced vaginal sex in the past 7 days and genital inflammation, we next examined whether consensual sex in the past 7 days might have a similar association, compared with those who had not had sex in the past 7 days at baseline (n=134; table 4), experience of any forced sex in the past 7 days remained significantly associated with genital inflammation (n=44; aOR=2.62, 95% CI 1.21 to 5.88, p=0.02), while having only consensual sex in the past 7 days was not (n=567; aOR=0.94, 95% CI 0.63 to 1.40, p=0.76).

**Table 4:** Associations between genital inflammation and consensual sex in the past 7 days (logistic regression model)

	Beta coefficient	aOR	95% CI	P value
Only consensual sex, past 7 days	−0.06	0.94	0.63 to 1.40	0.76
Any forced sex, past 7 days	0.96	2.62	1.21 to 5.88	**0.02**
Age	0.002	1.00	0.98 to 1.02	0.83
Medium SES	0 (reference)			
Low SES	−0.09	0.92	0.63 to 1.34	0.66
High SES	−0.08	0.92	0.63 to 1.34	0.67
Low Nugent score	0 (reference)			
Intermediate Nugent score	0.64	1.90	1.35 to 2.68	**<0.001**
High Nugent Score	−0.29	0.74	0.48 to 1.14	0.17
Vaginal cleaning (soap)	−0.28	0.75	0.41 to 1.35	0.34
Bacterial STI*	0.59	1.81	1.12 to 2.96	**0.02**
Low substance use	0 (reference)			
Medium substance use	−0.20	0.82	0.59 to 1.15	0.25
High substance use	−0.71	0.49	0.29 to 0.81	**<0.001**

Bold values denote statistical significance at the 5% level.

* Chlamydia, gonorrhoea or syphilis infection.

aOR, adjusted OR; SES, socioeconomic status; STI, sexually transmitted infection.

Finally, while data on forced vaginal sex more than 7 days ago were limited, we next investigated longer-term associations of genital inflammation among those with historical forced vaginal sex experience. Compared with those who had never been forced to have sex at baseline (n=342; table 5), forced sex exposure in the past 7 days remained significantly associated with genital inflammation (n=44; aOR=2.51, 95% CI 1.21 to 5.42, p=0.02), while forced sex exposure more than 6 months ago (n=130; aOR=0.70, 95% CI 0.65 to 1.34, p=0.11) and exposure within the past 6 months but not the past 7 days (aOR=0.93, 95% CI 1.21 to 5.42, p=0.70) were not.

**Table 5:** Associations between genital inflammation and non-recent forced vaginal sex exposure (logistic regression model)

	Beta coefficient	aOR	95% CI	P value
Forced sex exposure, over 6 months ago	−0.36	0.70	0.45 to 1.08	0.11
Forced sex exposure, past 6 months	−0.07	0.93	0.65 to 1.34	0.70
Forced sex exposure, past 7 days	0.92	2.51	1.21 to 5.42	**0.02**
Age	0.005	1.00	0.98 to 1.03	0.66
Medium SES	0 (reference)			
Low SES	−0.09	0.91	0.62 to 1.33	0.64
High SES	−0.07	0.93	0.64 to 1.35	0.70
Low Nugent score	0 (reference)			
Intermediate Nugent score	0.66	1.93	1.37 to 2.72	**<0.001**
High Nugent Score	−0.29	0.75	0.48 to 1.15	0.19
Vaginal cleaning (soap)	−0.28	0.76	0.41 to 1.37	0.35
Bacterial STI*	0.62	1.85	1.15 to 3.03	**0.01**
Low substance use	0 (reference)			
Medium substance use	−0.16	0.85	0.61 to 1.20	0.35
High substance use	−0.68	0.50	0.30 to 0.85	**0.01**

Bold values denote statistical significance at the 5% level.

* Chlamydia, gonorrhoea or syphilis infection.

aOR, adjusted OR; SES, socioeconomic status; STI, sexually transmitted infection.

## Discussion

Forced vaginal sex has consistently been associated with increased risk of HIV acquisition in epidemiological studies.^27 28^ While some of this increased acquisition risk is almost certainly due to demographic and behavioural factors that increase the risk of exposure to HIV, we were interested to understand whether forced vaginal sex might also increase biological (per-exposure) HIV susceptibility. In this large cohort of FSWs in Nairobi, we find that forced vaginal sex was very common, and that recent (past 7 days) forced sex exposure is associated with genital inflammation, as defined by a clinically validated composite score of proinflammatory cytokines that has been previously associated with HIV seroconversion. This inflammatory effect was not observed in women having recent consensual sex and was not apparent beyond 7 days of the forced sex exposure.

Interestingly, we did not demonstrate an association with our co-primary endpoint of sE-cad concentration, a validated biomarker of epithelial damage. Our survey tools provided limited granularity regarding the elapsed time since a participant had experienced forced vaginal sex. In our previous studies of vaginal soluble immune factor concentrations before and after consensual penile-vaginal sex,^29^ there was transient elevation of the biomarker of epithelial damage sE-cad with return to baseline by 72 hours; similar effects were observed for some inflammatory cytokines, but IL-8 levels remained elevated beyond 72 hours. Furthermore, our in vitro study of sE-cad release after physical disruption of endocervical cells demonstrated that epithelial trauma and inflammation were inextricably intertwined, with an immediate post-traumatic increase in sE-cad, followed by a more delayed increase in levels of the inflammatory cytokine IL-6.^14^ This suggests that our ability to see elevated biomarkers relating to epithelial damage would be substantially impaired if Maisha Fiti participants reporting recent forced sex exposure were exposed more than 72 hours prior to their study visit. Therefore, in this study, we may have captured the more prolonged release and/or increased production of proinflammatory cytokines, but have missed the associated but shorter-lived increase in sE-cad. Our subanalysis on historical forced vaginal sex suggests that more specific data on time-since-sex would be useful to understanding the temporality of mucosal immune responses following an assault.

To our knowledge, this is the largest study to date to investigate genital inflammation among women who have recently experienced forced vaginal sex. Given the considerable challenges and sensitivities inherent in the conduct of sexual assault research,^16^ few studies have investigated the effect of forced sex on biological HIV susceptibility. A previous pilot study of immune biomarkers in women who experienced sexual assault in the past 12 weeks (n=13) reported increased genital IL-1α and decreased genital MCP-1 and plasma MIP-3α in exposed women,^30^ although this may be due in part to the significantly different BV prevalences in exposed and unexposed women in this study, since BV has important immune effects and was not controlled for in the immune analysis. The size of our cohort (n=746) means that we were able to examine the effects of very recent forced vaginal sex on vaginal immune parameters, and also to confirm that the immune differences observed were independent of potential confounders such as altered vaginal flora.

Another recent exploratory study of FSWs in Mombasa^31^ found no association between recent sexual violence (defined as forced vaginal, anal or oral sex 30 or fewer days ago) and IFN-γ, TNF, IL-1β, MIP-3α, MIP-1β or IP-10 concentrations, though the authors did find an association between previous sexual violence experience (forced vaginal, anal or oral sex more than 30 days ago) and IL-10. These relationships did not change in a sensitivity analysis where recent sexual violence was redefined as forced vaginal, anal or oral sex in the past 7 days. While this study had limited power to detect immunological differences (25/285 participants experienced forced sex 30 or fewer days ago), it highlights the methodological differences in biological sampling (eg, menstrual cup, cervicovaginal lavage, vaginal swabbing), definition of sexual violence (eg, timing of recent exposure, whether forms of sexual violence besides sexual assault are included) and biological measures (eg, number of cytokines assessed, use of a composite score) that make comparisons between studies in this field difficult.

In our study, participants who self-reported forced vaginal sex in the past 7 days were more likely to be experiencing poverty, depression and high-risk substance use. PrEP uptake across the entire cohort was low, and very few women accessed PEP after their assault. Importantly, despite recently experiencing forced vaginal sex, these participants had met with a median of 4 clients in the past 7 days, clearly demonstrating that during this window of heightened vulnerability to HIV acquisition, many women will continue to solicit clients in order to provide for themselves and their children, parents and partners. This could also suggest that many women in this cohort who are sexually assaulted are also engaged in survival sex, as defined by the exchange of sex for payment in money, drugs or shelter as a consequence of poverty and economic dependence.^32^ This further highlights the elevated HIV risk that lies at the intersection of gender-based violence, substance use and poverty, and emphasises that it will be key to develop strategies that enable the provision of effective HIV prevention tools, appropriate counselling and other services to FSWs during the period immediately following sexual assault.

Our study has some limitations. Measurements of some soluble immune factors using the MSD multiplex system were limited by the range of quantification for each analyte: in particular, the saturated signal we measured for sE-cad in many participants suggests that levels may have been well above the limit of quantification in some participants. Specific details of the type of forced sex exposure were not collected, and due to the wording of the interview question about sexual assault, it is possible that some participants were exposed to forced oral or anal sex and not vaginal sex. Since the participants of the Maisha Fiti study were all existing clients of SWOP clinics, they may possess sociobehavioural and sexual health characteristics distinct from FSWs not enrolled in such clinics, potentially introducing selection bias. As a sensitive topic, recent violence experience may have been under-reported in this study, and women experiencing ongoing violence may be less likely to participate in a research study, especially if the perpetrator is an intimate partner. Therefore, the data presented here may be an underestimate of the true prevalence of recent forced sex exposure in this population, as well as the true effect of forced sex on genital immune parameters. However, we attempted to reduce the risk of this reporting bias through strict confidentiality and data protection practices: specifically, the Maisha Fiti study was advertised as a women’s health study (not a violence study) to reduce the risk of further violence from boyfriends or partners; women were asked to attend the study clinic alone or with a trusted female friend; interviews took place in a private space where conversations could not be overheard; and questionnaires and lab samples were identifiable by a unique participant number only. Finally, although the nine cytokine/chemokine immune parameters measured were selected based on previous research, the number of immune parameters was relatively limited due to the large sample size and assay cost. A more complete characterisation of the genital immune microenvironment after forced sex exposure, including analysis of sex hormone levels, genital immune cell populations and anti-inflammatory cytokine data, and further elucidation of the exact duration of inflammation after sexual assault, would be important to consider for future studies.

Overall, this is the first large-scale study to assess the impact of recent forced vaginal sex exposure on genital inflammation and other immune correlates of HIV susceptibility. Women recently exposed to forced vaginal sex, but not to consensual vaginal sex, were more likely to demonstrate genital inflammation in the week following forced vaginal sex, a period during which most women continue to meet with clients. This underscores the importance of further integration of HIV and GBV services,^33^ particularly through the involvement of peer networks and community leadership, which were a significant strength of the Maisha Fiti study. Violence-specific capacity building, as well as enhanced PrEP access and referral pathways for women experiencing GBV, should also be considered as strategies for tackling the HIV/GBV syndemic.

## Supplementary material

10.1136/bmjgh-2024-018219 online supplemental file 1

## Data Availability

The data are not publicly available due to the need to protect participant confidentiality and safety. Our commitment to participant confidentiality and safety is detailed in our participant information sheet and consent forms, as approved by three ethics committees: Kenyatta National Hospital (P778/11/2018), The London School of Hygiene and Tropical Medicine (16229) and the University of Toronto (37046). Requests to access data should be directed to: researchdatamanagement@lshtm.ac.uk, citing item 3643 - The Maisha Fiti Study.
