# Supplementary material for: Forced vaginal sex and genital immune correlates of HIV risk: a prospective study of female sex workers in Kenya
Source: BMJ Glob Health. 2025 Dec 19;10(12):e018219. doi: 10.1136/bmjgh-2024-018219 (PMC12718569; doi:10.1136/bmjgh-2024-018219)
Supplement: online supplemental file 1 [file bmjgh-10-12-s001.docx]

**Supplementary file 1.**

**Table s1**. Description of exposure variables used in the Maisha Fiti study.

| Variable | Tool/Question | Indicator |
| --- | --- | --- |
| Forced vaginal sex | “Have you been physically forced to have sex in the past seven days?” | YES |
| Socioeconomic status | 14 household asset questions used in the Kenyan Demographic Health Surveys | Principle component analysis (PCA) used to compute household wealth tertiles: Low, Medium, High |
| Depression | Patient Health Questionnaire-9 (PHQ-9) | score ≥10 (moderate/severe depression) |
| Anxiety | Generalised Anxiety Disorder-7 Assessment (GAD-7) | score ≥10 (moderate/severe anxiety) |
| PTSD | Harvard Trauma Questionnaire (HTQ-17) | score ≥ 2.5 (positive for PTSD) |
| Harmful alcohol/substance use | WHO ASSIST (Alcohol, Smoking and Substance Involvement Screening Test) tool | Low risk 0–10; moderate risk ≥11; high risk >27 |

**Figure s1**. Associations between forced vaginal sex exposure in the past seven days and individual proinflammatory cytokines.
